# Rapid Sentinel Surveillance for COVID-19 — Santa Clara County, California, March 2020

**DOI:** 10.15585/mmwr.mm6914e3

**Published:** 2020-04-10

**Authors:** Marissa L. Zwald, Wen Lin, Gail L. Sondermeyer Cooksey, Charles Weiss, Angela Suarez, Marc Fischer, Brandon J. Bonin, Seema Jain, Gayle E. Langley, Benjamin J. Park, Danielle Moulia, Rory Benedict, Nang Nguyen, George S. Han

**Affiliations:** ^1^Santa Clara County COVID-19 Response Field Team, CDC; ^2^County of Santa Clara Public Health Department, San Jose, California; ^3^California Department of Public Health, Richmond, California; ^4^Palo Alto Medical Foundation, Palo Alto, California; ^5^Santa Clara Valley Medical Center, San Jose, California.

On February 27, 2020, the Santa Clara County Public Health Department (SCCPHD) identified its first case of coronavirus disease 2019 (COVID-19) associated with probable community transmission (i.e., infection among persons without a known exposure by travel or close contact with a patient with confirmed COVID-19). At the time the investigation began, testing guidance recommended focusing on persons with clinical findings of lower respiratory illness and travel to an affected area or an epidemiologic link to a laboratory-confirmed COVID-19 case, or on persons hospitalized for severe respiratory disease and no alternative diagnosis (*1*). To rapidly understand the extent of COVID-19 in the community, SCCPHD, the California Department of Public Health (CDPH), and CDC began sentinel surveillance in Santa Clara County. During March 5–14, 2020, four urgent care centers in Santa Clara County participated as sentinel sites. For this investigation, county residents evaluated for respiratory symptoms (e.g., fever, cough, or shortness of breath) who had no known risk for COVID-19 were identified at participating urgent care centers. A convenience sample of specimens that tested negative for influenza virus was tested for SARS-CoV-2 RNA. Among 226 patients who met the inclusion criteria, 23% had positive test results for influenza. Among patients who had negative test results for influenza, 79 specimens were tested for SARS-CoV-2, and 11% had evidence of infection. This sentinel surveillance system helped confirm community transmission of SARS-CoV-2 in Santa Clara County. As a result of these data and an increasing number of cases with no known source of transmission, the county initiated a series of community mitigation strategies. Detection of community transmission is critical for informing response activities, including testing criteria, quarantine guidance, investigation protocols, and community mitigation measures (*2*). Sentinel surveillance in outpatient settings and emergency departments, implemented together with hospital-based surveillance, mortality surveillance, and serologic surveys, can provide a robust approach to monitor the epidemiology of COVID-19.

During March 5–14, 2020, four urgent care centers in Santa Clara County were selected to participate as sentinel sites based on varied geographic locations throughout the county, diversity in adult and pediatric patient populations served by the centers, and staffing and resource capacity to collect and transport specimens. For this investigation, county residents evaluated with respiratory symptoms (e.g., fever, cough, or shortness of breath) who had no recent travel to an area outside the United States with sustained COVID-19 transmission and no known close contact with a patient with confirmed COVID-19 were identified at one of the four participating urgent care centers. Health care providers obtained a nasopharyngeal swab for influenza virus testing as part of routine clinical care and notified participants that their specimen might be tested for SARS-CoV-2. Because of limited testing capacity, a convenience sample of the first 5–10 specimens that tested negative for influenza virus each day were sent to the Santa Clara County Public Health Laboratory for SARS-CoV-2 testing using the CDC 2019-nCoV real-time reverse transcription–polymerase chain reaction assay (*3*). SARS-CoV-2 test results, age, and sex of each patient were reported to SCCPHD. Potential differences among patients who were and were not tested for SARS-CoV-2 could not be examined in this investigation.

During the investigation period, 226 patients seen at one of the four urgent care centers met the inclusion criteria (i.e., Santa Clara county resident, respiratory symptoms, no recent travel, and no known close contact with a patient with confirmed COVID-19) and were tested for evidence of influenza virus infection; among those, 53 (23%) had positive test results for influenza. Among the remaining 173 patients with negative test results for influenza, 79 specimens were tested for SARS-CoV-2; of those, nine (11%) had evidence of SARS-CoV-2 infection. Persons with positive test results for COVID-19 were adults with a median age of 46 years (range = 30–57 years); six (67%) were female. Among the 70 patients with negative SARS-CoV-2 test results, 51 (73%) were adults aged ≥18 years, and the median age was 31 years (range 6 months–81 years); 39 (56%) were female. Patients with positive test results for COVID-19 were notified and placed in isolation, case investigations and contact tracing were initiated, and positive test results were reported to CDPH and CDC.

## Discussion

Identification of cases from this sentinel surveillance system helped confirm community transmission of SARS-CoV-2 in Santa Clara County. Among county residents evaluated at participating urgent care centers in early March with respiratory illness and no known exposure to SARS-CoV-2, approximately one quarter had positive test results for influenza, but 11% of patients with negative test results for influenza had positive test results for COVID-19. If it is assumed there were no influenza and SARS-CoV-2 coinfections and that persons with negative test results for influenza and not tested for SARS-CoV-2 were similar to those who were tested, then an estimated 8% (19 of 226) of persons seen at participating urgent care centers with respiratory symptoms had COVID-19. This is similar to the 5% SARS-CoV-2 infection rate identified among patients evaluated for mild influenza-like illness at one Los Angeles medical center during a similar time frame (*4*).

The findings in this report are subject to at least two limitations. First, SARS-CoV-2 testing was performed on a convenience sample of specimens that tested negative for influenza. Second, the findings are based on a small number of patients evaluated for respiratory illness at four participating sentinel sites and might not be representative of the broader community. However, as a result of these data and an increasing number of cases with no known source of transmission in Santa Clara County, the county initiated a series of community mitigation strategies to slow the spread of SARS-CoV-2. On March 9, the county issued recommendations to cancel gatherings of ≥1,000 people and to take action to protect vulnerable populations (e.g., older adults).* On March 16, Santa Clara County and five adjacent counties joined to order all residents to shelter in place and all schools, businesses, and government agencies to cease nonessential operations (*5*). Santa Clara County also posted updated community mitigation guidance and recommendations for populations at high risk, long-term care facilities, and hospitals (*6*).

Early implementation of community intervention is likely essential to maximize its effectiveness in slowing the spread of SARS-CoV-2 (*2*). Local public health departments can use sentinel surveillance to assess the level of community transmission of COVID-19 and to better guide the selection and implementation of community mitigation measures, including the scale, timing, duration, and settings in which to focus these strategies (*7*). Sentinel surveillance in outpatient settings and emergency departments, implemented together with hospital-based surveillance, mortality surveillance, and serologic surveys, can provide a robust, multifaceted approach to monitor the epidemiology of COVID-19.

**Summary**

**What is already known about this topic?**

On February 27, 2020, Santa Clara County, California, identified its first case of coronavirus disease 2019 (COVID-19) associated with probable community transmission.

**What is added by this report?**

During March 5–14, among patients with respiratory symptoms evaluated at one of four Santa Clara County urgent care centers serving as sentinel surveillance sites, 23% had positive test results for influenza. Among a subset of patients with negative test results for influenza, 11% had positive test results for COVID-19.

**What are the implications for public health practice?**

COVID-19 cases identified through this sentinel surveillance system helped confirm community transmission in the county. Local health departments can use sentinel surveillance to understand the level of community transmission of COVID-19 and to better guide the selection and implementation of community mitigation measures.

## Footnotes

* https://www.sccgov.org/sites/phd/DiseaseInformation/novel-coronavirus/Pages/order-cancellation-mass-gatherings.aspx.
